# I222 Neuraminidase Mutations Further Reduce Oseltamivir Susceptibility of Indonesian Clade 2.1 Highly Pathogenic Avian Influenza A(H5N1) Viruses

**DOI:** 10.1371/journal.pone.0066105

**Published:** 2013-06-11

**Authors:** Jennifer L. McKimm-Breschkin, Susan Barrett, Pudjiatmoko, Muhammad Azhar, Frank Y. K. Wong, Paul Selleck, Peter G. Mohr, James McGrane, Mia Kim

**Affiliations:** 1 Commonwealth Scientific and Industrial Research Organisation, Materials Science and Engineering, Parkville, Victoria, Australia; 2 Commonwealth Scientific and Industrial Research Organisation, Australian Animal Health Laboratory, Geelong, Victoria, Australia; 3 Directorate of Animal Health, Directorate General of Livestock and Animal Health Services, Ministry of Agriculture, Jakarta, Indonesia; 4 Food and Agriculture Organization of the United Nations, Emergency Centre for Transboundary Animal Diseases, Jakarta, Indonesia; 5 World Organisation for Animal Health and Food and Agriculture Organization of the United Nations Global Network of Expertise on Animal Influenza, (OFFLU) Paris, France and Rome, Italy; 6 National Veterinary Services Laboratories, Ames, Iowa, United States of America; Centers for Disease Control and Prevention, United States of America

## Abstract

We have tested the susceptibility to neuraminidase inhibitors of 155 clade 2.1 H5N1 viruses from Indonesia, isolated between 2006–2008 as well as 12 clade 1 isolates from Thailand and Cambodia from 2004–2007 using a fluorometric MUNANA-based enzyme inhibition assay. The Thailand and Cambodian clade 1 isolates tested here were all susceptible to oseltamivir and zanamivir, and sequence comparison indicated that reduced oseltamivir susceptibility we observed previously with clade 1 Cambodian isolates correlated with an S246G neuraminidase mutation. Eight Indonesian viruses (5%), all bearing I222 neuraminidase mutations, were identified as mild to extreme outliers for oseltamivir based on statistical analysis by box plots. IC_50_s were from 50 to 500-fold higher than the reference clade 1 virus from Viet Nam, ranging from 43–75 nM for I222T/V mutants and from 268–349 nM for I222M mutants. All eight viruses were from different geographic locales; all I222M variants were from central Sumatra. None of the H5N1 isolates tested demonstrated reduced susceptibility to zanamivir (IC_50_s all <5 nM). All I222 mutants showed loss of slow binding specifically for oseltamivir in an IC_50_ kinetics assay. We identified four other Indonesian isolates with higher IC_50_s which also demonstrated loss of slow binding, including one virus with an I117V mutation. There was a minimal effect on the binding of zanamivir and peramivir for all isolates tested. As H5N1 remains a potential pandemic threat, the incidence of mutations conferring reduced oseltamivir susceptibility is concerning and emphasizes the need for greater surveillance of drug susceptibility.

## Introduction

Highly pathogenic avian influenza (HPAI) H5N1 remains endemic in at least six countries including China, Egypt, Indonesia, Viet Nam, Bangladesh and parts of India, and continues to impact livelihoods and poultry farming in several other countries in South East Asia [1]. While not yet capable of human to human transmission, concerns regarding potential emergence of a pandemic virus remain as millions of poultry are infected annually. Sporadic transmission from poultry to humans continues; as of February 2013, the largest numbers of human cases reported to WHO were from Indonesia, Egypt, and Viet Nam (n = 192, 169, and 123 respectively) [2]. The case fatality rates vary significantly across these three countries, ranging from 36% (60/169) in Egypt to 83% (160/192) in Indonesia. This variation may reflect delays in initiating treatment including antiviral therapy, but may also represent some inherent variability in the drug susceptibility of different isolates.

Two drugs are licensed globally for the treatment and prevention of influenza, zanamivir (Relenza) administered by oral inhalation, delivering high doses to the upper respiratory tract, and oseltamivir (Tamiflu) which is taken orally and disseminates systemically. A third drug, peramivir, is licensed in Japan and South Korea for intravenous administration, but it is still undergoing clinical trials elsewhere. These drugs target the neuraminidase (NA) enzyme, a surface glycoprotein of the influenza virus, and are effective against all strains of influenza due to the high degree of conservation at the NA active site. Oseltamivir is the drug of choice for treatment of H5N1 infected patients due to concerns regarding potential systemic infection. Oseltamivir is also the primary drug stockpiled globally for potential pandemics. All these drugs, classed as neuraminidase inhibitors (NAIs), are designed based on 2,3-dehydro-2-deoxy-N-acetylneuraminic acid (DANA) a transition state analogue of the sialic acid substrate. Zanamivir has a single modification of a C4-guanidinium group compared to DANA [3], while oseltamivir has both a C4-amino group, and a pentyl ether group replaces the glycerol side chain in DANA [4]. Peramivir has features of both inhibitors, with a C4-guanidinium group and a pentyl side chain [5]. Both oseltamivir and peramivir require structural rearrangements in the active site for high affinity binding. Residue E276 rotates to form a salt link to R224, creating the pocket which accommodates their hydrophobic side chains. Because of the structural changes required to accommodate the binding of these inhibitors, we had predicted resistance was more likely to arise against oseltamivir than zanamivir [6]. Widespread resistance to oseltamivir was demonstrated by the global spread of the oseltamivir resistant seasonal H1N1 influenza strain during 2007–9 [7]–[10].

Despite the millions of poultry infected with HPAI, routine surveillance for drug susceptibility has not been conducted on avian isolates with information primarily from small studies [11]–[15]. Analysis of NA gene sequences in public data bases has identified known NAI resistance mutations in HPAI H5N1 avian virus isolates. Sequence analysis revealed mixed populations of oseltamivir sensitive wild type viruses and some with the H274Y mutation conferring oseltamivir resistance from chickens, ducks and geese [16]. Virus with a N294S mutation was detected in ducks [17]. However, while sequencing approaches can detect mixtures of wild type and mutant populations, only known mutations can be detected [18]. In contrast, screening using the NA enzyme inhibition assay will detect phenotypic differences caused by any mutation, but the resistant population generally needs to be in excess over the wild type population to detect a shift in susceptibility [19]. The technique also requires level 3 biocontainment facilities for virus culture to produce sufficient material to be analyzed in the assay, which can be a barrier to large scale surveillance of drug susceptibility.

We previously evaluated a panel of clade 1 HPAI H5N1 viruses from Viet Nam and Cambodia and clade 2.1 viruses from Indonesia using a fluorometric 4-Methylumbelliferyl N-acetyl-α-D-neuraminic acid (MUNANA) based enzyme assay. We showed that the clade 2.1 viruses circulating in Indonesia had a lower susceptibility to oseltamivir with a mean IC_50_ of 11.5 nM (IC_50_ = concentration of drug to inhibit enzyme activity by 50%) compared to the clade 1 isolates with a mean IC_50_ of 0.5 nM [13]. We suggested that an H252Y difference in the NA between clade 1.1 and clade 2.1 respectively was the most likely cause of the lower oseltamivir susceptibility. This was subsequently confirmed by others using mutagenesis [20], [21]. The Y252 impairs the rotation of E276 in the NA active site, which is necessary to form the hydrophobic pocket to accommodate the pentyl ether side chain of oseltamivir. Susceptibility to zanamivir is not affected. The reduced susceptibility to oseltamivir *in vitro* also corresponds to reduced susceptibility in animal models [20], [22].

We also reported that some HPAI H5N1 viruses isolated from the Kandal province in Cambodia in 2005 had approximately 6–7-fold reductions in susceptibility to oseltamivir compared to the isolates from 2004, but there was no difference in zanamivir susceptibility. We did not know whether this was a regional cluster or whether this was more widespread [13]. Based on observations of Rameix-Welti et al. [15] we suggested that a S246G mutation may be associated with this reduced susceptibility, but sequence data was not available at that time. The availability of further isolates from Cambodia has now enabled us to test this hypothesis further.

We aimed to extend the scope of our previous study by evaluating the drug susceptibility of a large panel of HPAI H5N1 virus isolates from Indonesia from 2006–2008 as well as avian isolates from Cambodia and Thailand from 2004–2007. Samples were screened against oseltamivir and zanamivir using the fluorometric MUNANA-based enzyme assay. Based on box plot statistical analysis, those identified as outliers were also screened against peramivir. Overall, higher IC_50_s were confirmed for oseltamivir in clade 2.1 HPAI H5N1 viruses circulating in Indonesia compared to clade 1viruses. But more disturbingly, eight Indonesian viruses (5%), all bearing I222 mutations were identified statistically as mild to extreme outliers for oseltamivir. None of the H5N1 isolates tested demonstrated reduced susceptibility to zanamivir (IC_50_s all <5 nM).

## Results

We used samples selected from our previous work as control reference strains [13] for the enzyme inhibition assays. These included a clade 1.1 virus from Viet Nam (A/chicken/Vietnam/08/2004) which was sensitive to both zanamivir and oseltamivir, a clade 1 virus from Cambodia (A/goose/Kandal/2005) which had demonstrated a 6 to 7-fold reduction in oseltamivir susceptibility, and an Indonesian clade 2.1 virus A/chicken/Wates/126/2005, which had previously displayed around a 15-fold reduction in oseltamivir susceptibility compared to the clade 1.1 Viet Nam viruses we had tested [13].

### Susceptibility of H5N1 Viruses from Cambodia to NA Inhibitors

All Cambodian viruses isolated from 2005–2007 displayed high susceptibilities to oseltamivir, with similar low IC_50_s as seen for the clade 1.1 reference virus from Viet Nam (Table 1) and previous 2004 Cambodian isolates. Sequencing of the NAs of Cambodian isolates tested here and previously [13] revealed that all of the Kandal 2005 isolates previously identified with higher IC_50_s for oseltamivir had a S246G mutation. (Note due to differences in the length of the NA genes between avian and human N1 NAs N2 sequence numbering is used throughout the manuscript). This finding is in agreement with Rameix-Welti et al. [15] who demonstrated that an H5N1 NA with an S246G mutation had an eight-fold higher IC_50_ for oseltamivir. They proposed that the interaction of S246 with the pentyl ether side chain contributed to the affinity of the NA for oseltamivir. Three changes in the stalk common to these isolates, A45T, T51A, S61A (numbering based on the HPAI H5N1 stalk, due to difficulty in aligning H5N1 and N2 stalk amino acids because of insertions/deletions between them), and D402E in the NA head were unlikely to have affected the NA sensitivity of the 2005 Kandal viruses.

**Table 1:** Susceptibility of clade 1.1 HPAI H5N1 isolates from Cambodia to zanamivir and oseltamivir in the enzyme inhibition assay.

H5N1 Virus	ZanamivirIC_50_ nM^a^	Fold difference towild type	OseltamivirIC_50_ nM^a^	Fold difference towild type
A/chicken/Cambodia/CMB07.71LC3/2007	1.30	0.7	0.51	0.7
A/duck/Cambodia/CMB07.72/2007	1.28	0.7	0.65	0.9
A/chicken/Cambodia/CMB07.71LC4/2007	0.83	0.5	0.75	1.1
A/chicken/Cambodia/CMB07.71LC1/2007	0.99	0.6	0.77	1.1
A/chicken/Cambodia/CMB07.71LC2/2007	1.07	0.6	0.78	1.1
A/duck/Cambodia/CMB06.58/2006	1.43	0.8	0.81	1.1
A/chicken/Cambodia/CMB05.142/2005	1.53	0.8	1.35	1.9
**Mean (SD)**	**1.20 (0.25)** a		**0.80 (0.26)** a	
**Reference Clade 1.1 Strains**	**Mean (SD)**		**Mean (SD)**	
A/chicken/Vietnam/08/2004^b^	1.80 (0.73)^b^		0.71 (0.26)	
A/goose/Kandal/2005^c^	1.68 (0.8)		7.07 (1.33)	

a Samples were all assayed with a 30 min preincubation with inhibitor, and then MUNANA was added and reactions were stopped after 60 min and read. Samples were tested in duplicate and means were calculated from the log_10_ transformed values, then back transformed.

b A/chicken/Vietnam/08/2004 was used as the zanamivir and oseltamivir sensitive reference. Mean and standard deviation of six H5N1 assays carried out during the same period.

c Virus used as an elevated oseltamivir IC_50_ reference from previous testing. Mean and standard deviation of six H5N1 assays carried out during the same period.

### Susceptibility of H5N1 Viruses from Thailand to NA inhibitors

The NAI susceptibilities were determined for five avian clade 1.1 H5N1 viruses isolated from Thailand during 2004–2006 (Table 2). Surprisingly only one isolate, A/chicken/Suphanburi/2509/2004, had comparable susceptibility to the clade 1.1 Viet Nam reference virus (Table 2). The other four viruses from 2005–6 had approximately two-fold higher IC_50_s for zanamivir than observed with the Viet Nam reference virus or the sensitive Cambodian clade 1.1 isolates tested here (Table 1). Although several amino acid differences were detected between the NA sequences of the Cambodian and Thailand viruses, there was no consistent difference identified that could account for the slightly altered susceptibility. The A/chicken/Ayudhya/2057/2004 isolate which had the highest IC_50_ for both zanamivir and oseltamivir had two unique changes (P154S and I314L, N2 numbering) as compared to other clade 1.1 and clade 2.1 strains investigated here (except for A/chicken/West Java/Tja-31/2008 a clade 2.1 virus from Indonesia which also had P154S). In the region of P154 other amino acids including D151, R152 and R156 are known to be involved in substrate or inhibitor binding [23], [24] and mutations at these sites can reduce NAI susceptibility [25]. Hence a mutation at position 154 could impact on inhibitor binding. The IC_50_s for A/chicken/West Java/Tja-31/2008 for both inhibitors were still below 5 nM, suggesting that these mutations would be unlikely to have any clinical significance.

**Table 2:** Susceptibility of clade 1.1 HPAI H5N1 isolates from Thailand to zanamivir and oseltamivir in the enzyme inhibition assay.

H5N1 Virus	Zanamivir IC_50_ nM^a^	Fold difference towild type	OseltamivirIC_50_ nM^a^	Fold differenceto wild type
A/chicken/Suphanburi/2509/2004	1.9	1.1	0.61	0.9
A/chicken/Saraburi/10713/2005	3.5	1.9	0.68	1.0
A/chicken/Pichit/606988/2006	3.5	1.9	0.69	1.0
A/duck/Suphanburi/14376/2005	3.6	1.9	1.1	1.5
A/chicken/Ayudhya/2057/2004	4.7	2.6	1.8	2.5
**Mean (SD)**	**3.43 (1.0)** a		**0.97 (0.5)** a	
**Reference clade 1.1 strain**	**Mean (SD)**		**Mean (SD)**	
A/chicken/Vietnam/08/2004^b^	1.80 (0.73)^b^		0.71 (0.26)	

a Samples were all assayed with a 30 min preincubation with inhibitor, and then MUNANA was added and reactions were stopped after 60 min and read. Samples were tested in duplicate and means were calculated from the log_10_ transformed values, then back transformed.

b Virus used as zanamivir and oseltamivir sensitive reference. Mean and standard deviation of six H5N1 assays carried out during the same period.

### Susceptibility of H5N1 Viruses from Indonesia to NA Inhibitors

**Figure 1:**
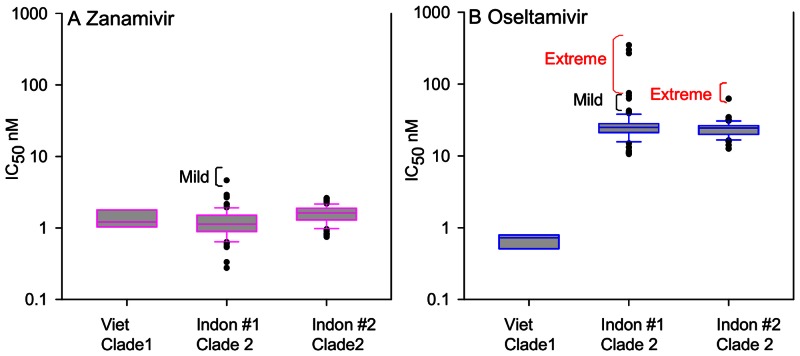
Box plots of means of IC_50_s for zanamivir and oseltamivir for Indonesian HPAI H5N1 isolates. Means were calculated from the log_10_ transformed duplicate values, then back transformed. Boxes represent the 25th to 75th percentiles, and horizontal lines within the boxes represent the median values. The difference between the 25^th^–75^th^ percentiles is defined as the interquartile range (IQR). The ends of the solid lines extending either side of the boxes represent the approximate 95% confidence limits. Mild and extreme outliers lie outside these 95% confidence limits at 1.5x or 3x the IQR respectively from the 75^th^ percentile. Viet clade 1 is the pooled results of all the assays using the reference clade 1.1 A/chicken/Vietnam/08/2004, Indon#1 = clade 2.1 Indonesian samples from batch 1, and Indon #2 = samples from batch 2. (A) Only one outlier was identified for zanamivir whereas there were 8 mild or extreme outliers for oseltamivir (B).

The virus samples from Indonesia were obtained in two batches over a year apart. The first contained 92 viruses primarily collected in 2007, and the second batch contained 63 viruses collected from 2006–2008. Due to the timing and large numbers of viruses, testing for each batch was carried out in multiple assays against both zanamivir and oseltamivir, but we used the same control reference viruses to enable comparison. For such large numbers of isolates, resistant viruses with elevated IC_50_s can distort the mean, hence box and whisker plots are used to demonstrate the median and the spread of IC_50_s, as well as to identify statistical outliers [26], [27] (Fig. 1). The summary of the statistical analysis is presented in Table 3.

**Table 3:** Susceptibility of clade 2.1 HPAI H5N1 isolates from Indonesia to zanamivir and oseltamivir in the enzyme inhibition assay.

H5N1 Virus	Zanamivir IC_50_ nM^a^Batch 1	Oseltamivir IC_50_ nM^a^Batch 1	Zanamivir IC_50_ nM^a^Batch 2	Oseltamivir IC_50_ nM^a^Batch 2
Number of viruses tested	91	92	63	63
Mean^b^ (range)	1.15 (0.28–4.67)	25.1^c^(10.6–349)	1.55 (0.75–2.62)	23.3(12.6–62.4)
Median	1.12	24.9	1.6	24.4
IQR^d^	0.89–1.51	21.2–28.2	1.3–1.9	20.4–26.4
Mild High Outliers^e^	1 (>3.3)	2 (>42.4)	0 (>3.2)	0 (>40.0)
Extreme High Outliers^f^	0 (>7.2)	5 (>63.7)	0 (>5.6)	1 (>60.5)
**Reference Controls ** [**13**]	**Mean (SD)**	**Mean (SD)**	**Mean (SD)**	**Mean (SD)**
Cl 1.1 A/chicken/Vietnam/08/2004^g^	1.80 (0.73)	0.71 (0.26)	2.80 (0.89)	0.84 (0.21)
Cl 1.1 A/goose/Kandal/2005^h^	1.68 (0.8)	7.07 (1.33)	2.94 (0.73)	6.55 (1.10)
Cl 2.1 A/chicken/Wates/126/2005^i^	0.92 (0.35)	15.9 (4.03)	1.61 (0.37)	19.80 (4.73)

a Samples were all assayed with a 30 min preincubation with inhibitor, and then MUNANA was added and reactions were stopped after 60 min and read. Samples were tested in duplicate and means and SDs for reference controls are from all assays in each batch, with six assays in the first batch and five in the second.

b Means for each batch were calculated on log_10_ transformed data, and then back transformed.

c Excluding 2 most extreme outliers with IC_50_s >260 nM.

d IQR Interquartile range representing the range in which 50% of values fall (25^th^-75^th^ percentile).

e Mild outliers defined as 1.5xIQR from the 75^th^ percentile.

f Extreme outliers defined as 3xIQR from the 75^th^ percentile.

g Clade 1 strain sensitive to both zanamivir and oseltamivir.

h Clade 1 Cambodian strain with small reduction in oseltamivir sensitivity.

i Clade 2 Indonesian strain with higher reduction in oseltamivir sensitivity.

All of the Indonesian clade 2.1 viruses displayed elevated IC_50_s to oseltamivir as compared to the clade 1.1 reference virus from Viet Nam and the other clade 1.1 viruses tested here from Cambodia and Thailand (Tables 1 and 2). The median IC_50_ was 25 nM with 65% (100/155) with IC_50_s between 20 and 30 nM, and 15% (24/155) had values over 30 nM (Fig. 1). Based on the statistical definition of mild and extreme outliers as discussed in the Materials and Methods we identified two mild outliers for oseltamivir with IC_50_s between 42 and 63 nM and five extreme outliers with IC_50_s between 63–350 nM (Table 4) in the first batch and one extreme outlier in the second batch with an IC_50_ of 62.4 nM. The IC_50_s of even the mild outliers to oseltamivir were more than 50-fold higher than the reference Viet Nam clade 1.1 IC_50_ with the most extreme outlier being up to 500-fold higher. While there was one outlier to zanamivir in batch one with an IC_50_ of 4.7 nM, this is less than three-fold higher than the reference Viet Nam virus, and is considered within the normal susceptible range for human isolates [26]. This isolate displayed no reduced susceptibility to oseltamivir.

**Table 4:** IC_50_s in the enzyme inhibition assay of clade 2.1 Indonesian viruses identified as outliers and associated sequence changes.

H5N1 Virus	Zanamivir IC_50_ nM^a^	Fold Difference^b^	Oseltamivir IC_50_ nM^a^	Fold Difference^b^	NA Sequence change
A/chicken/Tabanan/BBVD-307/2007	4.7	2.6	11.8	16.6	V263I
A/chicken/Bangli/BBVD-562/2007	2.9	1.6	36.0	50.7	I117V
A/chicken/Pidie/BPPVRI-15/2007	2.8	1.6	42.5	59.9	I222T
A/chicken/Tabanan/BBVD-142/2007	1.7	0.9	63.2	89.0	I222T
A/chicken/Denpasar/BBVD-456/2007	2.2	1.2	68.9	97.0	I222T
A/chicken/Tabanan/BBVD-107/2007	1.7	0.9	75.1	105.8	I222T
A/chicken/Kuantan Singingi/BPPVRII-620/2007	0.7	0.4	268	377.5	I222M
A/chicken/Padang Panjang/BPPVRII-272/2007	1.9	1.1	349	491.5	I222M
A/chicken/Siak/BPPVRII-635/2007^c^	ND	–	ND	–	I222M
A/Muscovy duck/Magelang/BBVW-415/2007	2.6	1.4	62.4	87.9	I222V
**Reference strain**	**Mean (SD)**		**Mean (SD)**		
A/chicken/Vietnam/08/2004^b^	1.8 (±0.73)		0.71 (±0.26)		

a Samples were all assayed with a 30 min preincubation with inhibitor, and then MUNANA was added and reactions were stopped after 60 min and read. Values are the mean of duplicate reactions.

b Fold difference was compared to the means of the six batch 1 assays for the sensitive reference clade 1.1 A/chicken/Vietnam/08/2004.

c Sample not inhibited by either drug; co-infected with Newcastle disease virus.

The NA sequences of all 155 clade 2.1 viruses (batches 1 and 2) were aligned to determine whether those isolates identified as mild or extreme outliers for either drug had any known mutations (Table 4). The mild outlier to zanamivir A/chicken/Tabanan/BBVD-307/2007 had a unique V263I change compared to other clade 2.1 isolates tested here. The I263 is also present in most of the clade 1.1 Cambodian strains tested here, but their IC_50_ values were 1.5 nM or less for zanamivir. However, as multiple differences exist between the clade 1 and clade 2.1 NA sequences, it is possible that a given mutation could have an impact on one clade but not the other.

Overall for oseltamivir, there were four mild or extreme outliers with I222T NA mutations, one extreme outlier with an I222V mutation and three extreme outliers which had I222M NA mutations (Table 4). One virus with the I222M mutation (A/chicken/Siak/BPPVRII-635/2007) was not inhibited by either drug, which is especially unusual for zanamivir. Based on previous experience this suggested the isolate was a co-infection of H5N1 with Newcastle disease virus (NDV), which also has NA enzyme activity. NDV was subsequently also identified in this sample. This should be noted as a warning to those carrying out NAI susceptibility testing on avian isolates that NDV can lead to incorrect interpretations of the presence of highly resistant influenza viruses. In this case there was coincidentally a virus with an I222M mutation which does cause high level oseltamivir resistance. All I222M variants were from central-Sumatra and two had identical hemagglutinin (HA) and NA sequences. The NAs of the three viruses with I222T mutations from Denpasar and Tabanan, Bali all had identical amino acid sequences including a V321I not seen in any of the other isolates here, as well as an I8M (H5N1 stalk numbering) and S48L (H5N1 stalk numbering) in the stalk region. There were however several nucleotide differences. The fourth I222T virus was isolated from Pidie, which is geographically distant (north end of Sumatra) from where the other I222T mutant viruses were isolated, and bore two additional amino acid changes. The A/Muscovy duck/Magelang/BBVW-415/2007 virus which was an extreme outlier to oseltamivir with an IC_50_ of 62.4 nM (Table 4), originated from Java. Sequence analysis revealed its NA had several variations in the stalk region compared to all other isolates, and it had a unique mutation of I222V.

Mutations leading to resistance to the NAIs often lead to a loss of fitness of the mutant virus, which may be outgrown by a wild type virus upon culturing. Close inspection of the sequencing chromatographs of all the I222 mutants showed no evidence of mixed sequences of wild type and mutant at position 222 in any of the isolates. This indicated that these mutant viruses were sufficiently fit to compete with the wild type virus in the birds and/or after egg culture to become the dominant species in our samples.

We then analyzed sequences for unique variations which had previously been associated with altered NAI susceptibility and then cross-checked their IC_50_s. Sequence analysis of the NA of A/chicken/Bangli/BBVD-562/2007 revealed an I117V mutation (Table 4). There have been reports of I117V conferring reduced susceptibility to oseltamivir during surveillance of other H5N1 viruses [11], [12], [28], [29]. This virus had an IC_50_ in the higher range for each drug (zanamivir 2.9 nM, oseltamivir 36 nM). While not a mild outlier statistically, these values were well above the median for oseltamivir (25 nM).

### Analysis of NA Inhibitor Binding by IC_50_ Kinetics

The NAIs are described as slow binding inhibitors, and loss of slow binding is seen with many resistant viruses [30]. While laboratories routinely use a single time point for measuring the IC_50_, we have developed real time IC_50_ kinetics assays which additionally demonstrate whether an inhibitor is fast or slow binding by comparing the IC_50_s over 60 min with and without preincubation of NAI and virus [30]. If the NAI is slow binding, preincubation with virus is needed for maximum binding. Hence the final IC_50_ without preincubation is higher than the initial IC_50_ with preincubation. If the final IC_50_s for both sets of reactions are similar (ratio of ∼1) this indicates changed kinetics due to fast binding and dissociation or a partial loss of slow binding and faster dissociation of the NAI.

IC_50_ kinetics for the mutant viruses were compared to clade 1.1 and clade 2.1 reference controls, A/chicken/Vietnam/08/2004 and A/chicken/Bangli/BBVD-563/2007 respectively. This latter strain was used as the reference due to depletion of the A/chicken/Wates/126/2005 virus by the time these assays were carried out. It was considered a representative virus of the batch with an IC_50_ around the median.

**Figure 2:**
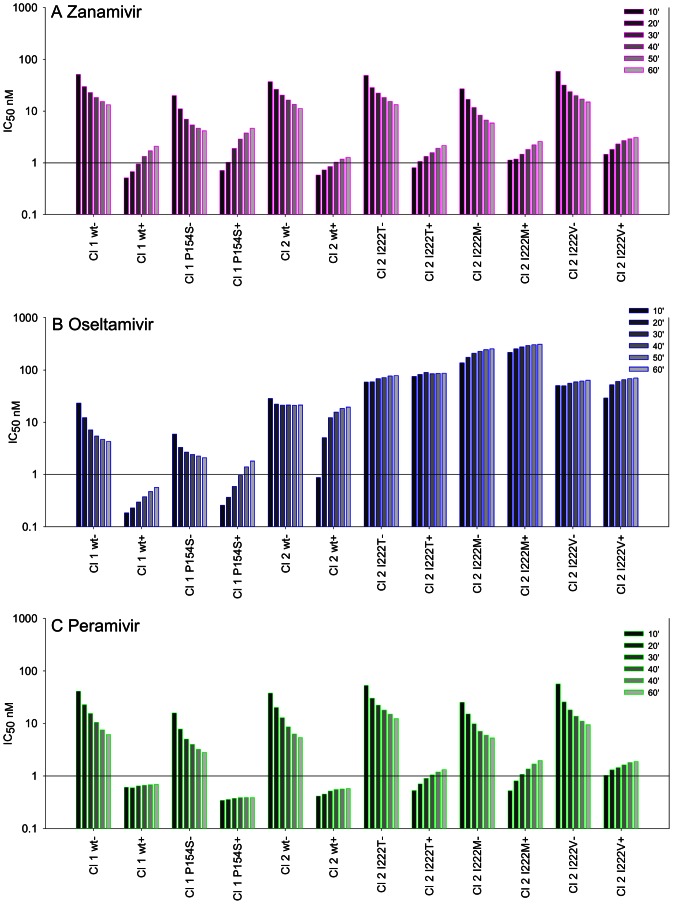
IC_50_ kinetics for wild type and mutant H5N1 isolates for zanamivir, oseltamivir and peramivir. Comparison of IC_50_s after each 10 min without preincubation of virus with inhibitor (−) and with a 30 min preincubation (+) of virus with inhibitor. After addition of MUNANA substrate both assays were incubated for 60 min. Results for each 10 min interval are the means of duplicate assays. A lower initial 10 min IC_50_ in the (+) reaction compared to the final 60 min IC_50_ in the (−) reaction indicates slow binding, e.g. all NAIs with the clade 1 wild type, (A) zanamivir, (B) oseltamivir and (C) peramivir. Similar IC_50_s in both assays demonstrate a loss of slow binding, e.g. all the I222 mutants with oseltamivir (B). A greater increase in IC_50_ from 10–60 min in the (+) reaction relative to the control virus indicates faster dissociation of the inhibitor compared to the wild type, e.g. clade 2.1 wild type with oseltamivir (B). **Cl 1 wt** = Clade 1 wild type A/chicken/Vietnam/08/2004, **Cl 1** **P154S** = clade 1.1 A/chicken/Ayudhya/2057/2004, **Cl 2 wt = **Clade 2.1 wild type A/chicken/Bangli/BBVD-563/2007, **Cl 2 I222T** = clade 2.1 A/chicken/Denpasar/BBVD-456/2007, **Cl 2 I222M** = clade 2.1 A/chicken/Padang Panjang/BPPVRII-272/2007, **Cl 2 I222V** = clade 2.1 A/Muscovy duck/Magelang/BBVW-415/2007.

For all wild type and mutant viruses for zanamivir and peramivir the final IC_50_s without preincubation were all higher than the initial IC_50_s with preincubation, indicating slow binding (Fig. 2). Ratios of the final IC_50_s were also all >2.0 (Table 5), except for the A/chicken/Ayudhya/2057/2004 clade 1 isolate with the P154S and I314L mutation with zanamivir and also with oseltamivir (ratio ∼1). Inspection of the graphs (Fig. 2) shows that the increased IC_50_s for this virus correlated with a more rapid dissociation of these two NAIs. A small increase in dissociation rates for zanamivir and peramivir was also observed for the I222 mutants.

**Table 5:** Comparison of 60 min IC_50_ values for enzyme inhibition assays with and without preincubation with inhibitor for wild type and mutant viruses.

		Zanamivir IC_50_ nM			Oseltamivir IC_50_ nM			Peramivir IC_50_ nM		
		Preincubation step^a^								
H5N1 virus^a^	NA Mutation	(−)	(+)	Ratio (−)/(+)	(−)	(+)	Ratio (−)/(+)	(−)	(+)	Ratio (−)/(+)
**Clade 1.1**										
A/chicken/Vietnam/08/2004	**Wild type**	13.2	2.1	6.3^b^	4.3	0.6	7.2^b^	6.2	0.7	8.9
A/chicken/Ayudhya/2057/2004	**P154S**	4.2	4.7	0.9^c^	2.1	1.8	1.2^c^	2.8	0.4	7
**Clade 2.1**										
A/chicken/Bangli/BBVD-563/2007	**Wild type**	11.2	1.3	8.6	21.4	19.6	1.1	5.4	0.6	9
A/chicken/Denpasar/BBVD-456/2007	**I222T**	13.3	2.2	6.0	78.2	86.1	0.9	12.4	1.3	9.5
A/chicken/Padang Panjang/BPPVRII-272/2007	**I222M**	5.9	2.6	2.3	254.0	310.3	0.8	5.3	2.0	2.7
A/Muscovy duck/Magelang/BBVW-415/2007	**I222V**	15.1	3.1	4.9	63.7	70.1	0.9	9.4	1.9	4.9

a (−)Virus, inhibitor and MUNANA substrate were added simultaneously with no preincubation. (+) virus and inhibitor were preincubated for 30 min, then MUNANA was added. Both reactions were followed for 60 min. Values are the means of duplicate reactions.

b Slow binding is demonstrated by a higher IC_50_ without preincubation compared to with preincubation; ratio of (−)/(+) >2.0.

c (−)/(+) ratio ∼1 shows changed kinetics, which can be due to fast binding and fast dissociation, as seen for all the I222 mutants with oseltamivir, or slow binding, but fast dissociation as seen for the P154S mutant with zanamivir and oseltamivir, as shown in Fig. 2.

For oseltamivir binding to the Indonesian reference virus, the final IC_50_ without preincubation was higher than the initial IC_50_ with preincubation, but the final ratio of the two reactions was ∼1 (Table 5). This confirmed our previous observation of partial loss of slow binding, and faster dissociation compared to the clade 1.1 virus, which had a 7-fold higher IC_50_ without preincubation [13]. The I222/M/V/T mutations all led to complete loss of slow binding of oseltamivir. Similar high IC_50_s were seen with or without preincubation with oseltamivir (ratio ∼1 Table 5).

**Figure 3:**
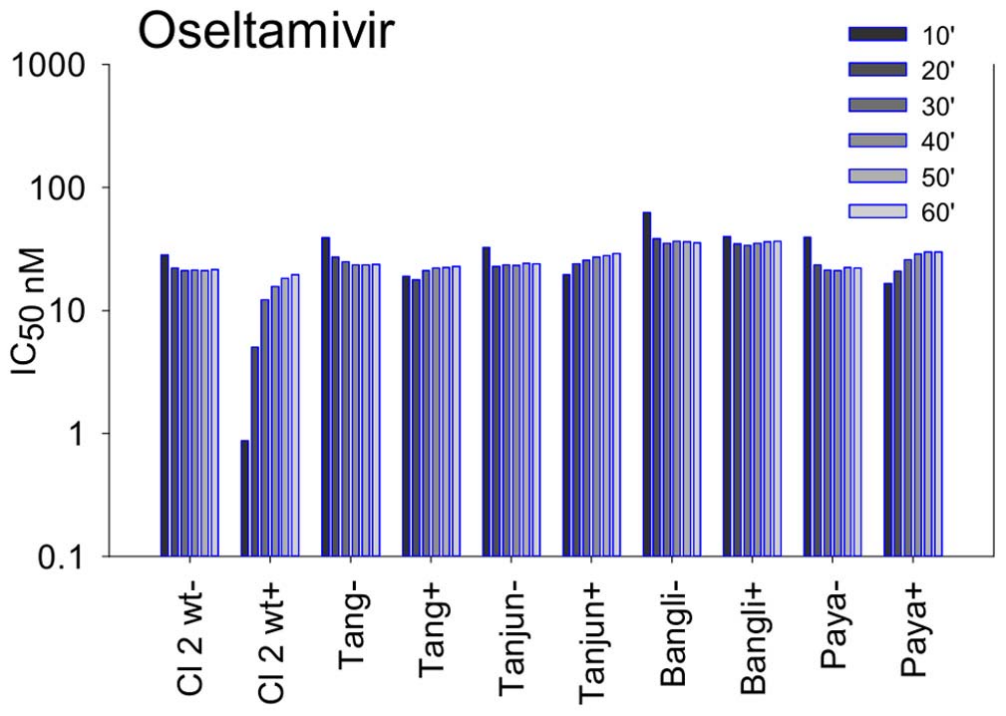
Identification of additional clade 2.1 viruses with altered oseltamivir binding by IC_50_ kinetics. Comparison of IC_50_s after each 10 min without preincubation of virus with inhibitor (−) and with a 30 min preincubation (+) of virus with inhibitor. After addition of MUNANA substrate both assays were incubated for 60 min. Lower initial 10 min IC_50_s in the (+) reaction compared to the final 60 min IC_50_s in the (−) reaction indicates slow binding. Similar IC_50_s in both assays demonstrate both fast binding and dissociation. These four isolates all demonstrated further loss of slow binding compared to the wild type clade 2.1 reference virus, although only one had a known mutation conferring reduced oseltamivir susceptibility. **Cl 2 wt** = Clade 2.1 wild type A/chicken/Bangli/BBVD-563/2007, **Tang** = A/chicken/West Java Tangerang/PTB6/2008, **Tanjun = **A/chicken/West Java/Tja-31/2008, **Bangli** = A/chicken/Bangli/BBVD-562/2007 (I117V mutation), **Paya** = A/chicken/Payakumbuh/BPPVRII-307/2007.

While the box plots identified Indonesian outliers with I222 mutations, because the median IC_50_ is already almost 30-fold higher than the sensitive clade 1.1 mean IC_50_s this approach does not identify all viruses which could be more resistant compared to sensitive clade 1.1 viruses. We therefore analyzed the kinetics of binding of the inhibitors for those viruses with IC_50_s around 30 nM to see if we could identify additional viruses with altered kinetics of NAI binding [30]–[32]. We identified four additional isolates which were no longer slow binding (Fig. 3). The first of these was A/chicken/Bangli/BBVD-562/2007, which correlated with the I117V mutation. The second isolate, A/chicken/West Java Tangerang/PTB6/2008 had unique S189G, E258K and G385E mutations. None of these are highly conserved structural or functional residues however, there was clearly an impact on the kinetics of oseltamivir binding. The third isolate A/chicken/West Java/Tja-31/2008 had three unique mutations compared to other clade 2.1 isolates tested, P154S, M257I, M306I. Interestingly we had observed a P154S in one clade 1.1 Thailand isolate which had a small impact on drug binding, so this may be the contributing mutation here, which in addition to the H252Y leads to complete loss of slow binding. The fourth isolate A/chicken/Payakumbuh/BPPVRII-307/2007 had no unique variation, hence there must be a combination of amino acids altering its kinetics of drug binding. Thus it appears that a number of variations can lead to loss of slow binding, other than those in known sites. None of these viruses demonstrated loss of slow binding to zanamivir or peramivir.

## Discussion

The importance of surveillance of the drug susceptibilities of human influenza isolates has led to widespread testing of NAI susceptibility of circulating human influenza strains in recent years, especially since the emergence of the oseltamivir resistant H1N1 seasonal strain in 2008 [7]–[10]. There are still concerns that a variant HPAI H5N1 strain may yet cause a pandemic, hence knowledge of the spectrum of drug susceptibility of H5N1 viruses is also critical for the management of patients infected with H5N1, as well as for stockpiling strategies for a potential pandemic arising from an H5N1 variant. There has been limited availability of avian influenza isolates for testing of drug susceptibilities, although even in those few tested there have been reports of decreased susceptibility of clade 2 isolates compared to clade 1 isolates [13], and additional mutations which have affected susceptibility including I117V, V116A, S246N, S246G and I222L [11], [12], [15].

In collaboration with the Indonesian Ministry of Agriculture, CSIRO AAHL has been involved with an FAO-implemented OFFLU technical project monitoring HA genetic and antigenic changes in Indonesian clade 2.1 HPAI H5N1 isolates. In addition to the information on the effects of HA drift mutations on antigenicity, these viruses provided a valuable opportunity for screening for susceptibility to the NAIs. The HPAI H5N1 viruses were from both commercial and backyard poultry, and covered different geographic areas of Indonesia. We also performed assays on a few virus samples from Cambodia and Thailand. Testing 166 HPAI H5N1 isolates in the MUNANA based enzyme inhibition assay detected no virus with an IC_50_>5 nM for zanamivir. In contrast we saw a higher mean and median IC_50_ for oseltamivir for the Indonesian isolates (∼25 nM) than in our previous report (11 nM). Of more concern is that we identified a number of clade 2.1 isolates from Indonesia which were phenotypically and genotypically resistant to oseltamivir. These mutations also resulted in different binding properties in our IC_50_ kinetics assays, with loss of slow binding to oseltamivir, a feature seen with many resistant NAs [30].

Although the H274Y mutation, which confers resistance to oseltamivir, is the most commonly detected mutation in influenza N1 viruses, there are increasing numbers of reports of mutations at residue I222 (I223 N1 numbering) including I222T/V/M/L conferring reduced NAI susceptibility in pandemic H1N1, seasonal H1N1 and H5N1 influenza strains. We identified five viruses with I222T or I222V mutations with IC_50_s in the 40–75 nM range, and three with I222M mutations, two with IC_50_s greater than 250 nM. Unlike the H274Y which only confers resistance in the N1 subtype, I222 mutations cause reduced susceptibility in N1 [11], [27], [33]–[40], N2 [41] and influenza B [27], [42]–[44] NAs, and have been detected after oseltamivir treatment in patients and *in vitro* exposure, but also spontaneously without drug exposure. Of approximately 1500 highly pathogenic H5N1 NA sequences in the NCBI Influenza Virus Resource, we found four I222V, four I222L, seven I222T, one I222Q and one I222M, an incidence of 1%. All mutations were found in clade 2 strains, with only the I222T found in clade 1 sequences. Although mutations at I222 generally only reduce susceptibility by less than 20-fold, what is of more concern is that they act synergistically to increase resistance to very high levels with H274Y [35], [37], [41] and E119V mutations. Our results suggest that the I222 mutations may also act synergistically with the H252Y mutation in the clade 2 viruses. Hurt et al. [35] generated I222V and I222M H5N1 mutants in a clade 1 background, and IC_50_s in their MUNANA based assay were 5 and 27 nM respectively. In contrast ours were 10-fold higher than these, with an IC_50_ greater than 250 nM for the I222M viruses, indicating that H252Y difference in the clade 2.1 background sequence also contributed to the higher IC_50_s. Interestingly the I222M mutation only had a small impact on peramivir binding, which also requires reorientation of E276 to form the hydrophobic pocket to accommodate its side chain [32], [45]. However even the H274Y mutation has less impact on peramivir binding compared to oseltamivir [30]. Similarly the H252Y difference between clade 1 and 2 viruses had a minimal effect on peramivir binding. This lack of effect may be due to the additional strong interactions of the 4-guanidinium group on peramivir.

We identified a virus with an I117V mutation, which although statistically was not an outlier, its IC_50_ for oseltamivir was well above the median IC_50._ There are more than 40 H5N1 viruses in the public sequence data bases with this mutation, and others have reported the spontaneous emergence of this mutation in H5N1 viruses from infected untreated ferrets [34] as well as in oseltamivir treated ferrets [46]. While others have reported I117V only confers a small difference in susceptibility in H5N1 isolates [11], [12], [28], [47], it has recently been demonstrated that the I117V acts synergistically with the H274Y mutation to increase oseltamivir and peramivir resistance to levels that would be of clinical concern [48]. Our results demonstrated that this mutation also affected the kinetics of oseltamivir binding compared to the wild type, hence it also appears to be acting synergistically with the H252Y mutation to further decrease oseltamivir susceptibility. We identified three additional isolates with IC_50_s in the 30 nM range which also demonstrated loss of slow binding. None of these had known mutations conferring altered susceptibility or mutations in conserved residues, but this demonstrates that there may be numerous changes which can also act synergistically with the H252Y to further reduce efficacy of oseltamivir binding.

We demonstrated that the decreased susceptibility to oseltamivir seen in some Cambodian isolates correlated with an S246G mutation, in agreement with Rameix-Welti et al. [15]. Residue 246 is reported to mediate hydrogen bonded interactions with the substrate and inhibitors [49]. There was a recent report that the S246G mutation had no impact on oseltamivir susceptibility in Cambodian isolates [14]. However they used the chemiluminescent assay, and relative drug susceptibility can differ between this and the MUNANA based assay we used [26]. An S246N mutation was also reported to cause a 24-fold reduction in oseltamivir susceptibility in isolates from Laos [11]. Fortunately the S246G mutation that we saw in our 2005 isolates appeared to be a limited cluster.

While the resistant viruses here were identified by statistical analysis, compared to the wild type clade 1.1 reference virus some of these viruses were between 50- and 500-fold resistant, but only 2- to 20-fold resistant to the clade 2.1 reference virus. However, there is no consensus on a definition of resistance that is known to relate to clinical failure. Many laboratories use a 10-fold change in IC_50_ in the enzyme inhibition assay compared to the wild type IC_50_, however since some viruses have a higher base line IC_50_, e.g. influenza B [26] and the clade 2.1 HPAI H5N1 strains, such viruses may be clinically resistant with only a few fold increase in IC_50_ compared to their wild type counterparts. The IC_50_ values also vary between the chemiluminescent and fluorescent enzyme inhibition assays [19], [26]. Furthermore our IC_50_ kinetics experiments demonstrate how the IC_50_ can change with incubation times in the NAI assays. However, for the drugs to have some therapeutic benefit the levels *in vivo* would need to be significantly higher than the IC_50_s. The levels of oseltamivir in plasma are estimated to be in the range from 400 to 1200 nmol/L [50], [51]. Levels in saliva are estimated to be less than 5% of plasma levels [52]. Thus levels in the upper respiratory tract may be significantly lower than 100 nM. With IC_50_s for oseltamivir for many of these outliers detected here >50 nM, they could present an even greater challenge for effective treatment with oseltamivir.

There are nine conserved residues in the NA active site which contact the sialic acid substrate, and a further ten residues which provide structural stability to these residues [23], [53]. Mutations conferring altered susceptibility to the NAIs have been mostly located within these amino acids [25]. However, the more the effects of mutations on the function of the NA are analyzed, the more it is obvious that non-active site residues can have subtle, but important effects on the enzyme function and stability. This was observed recently when an oseltamivir-resistant A/Brisbane/59/2007 like H1N1 virus emerged with an H274Y mutation, which rapidly spread globally, demonstrating no loss of fitness compared to the wild type virus. It has been demonstrated that three mutations (R222Q, V234M, D344N) compensated for the impact on fitness of the H274Y mutation [54], [55]. Our demonstration of differences in the kinetics of oseltamivir binding in NAs with no previously identified mutations emphasizes the need for phenotypic surveillance to detect the subtle effects of drift mutations on NAI susceptibility.

Since the emergence of the pandemic H1N1/09 virus there appears to be more complacency about the pandemic potential of HPAI H5N1 strains. However HPAI H5N1 viruses continue to spread and evolve and surveillance is of critical importance to inform on both the antigenic variation for vaccine preparation, as well as their antiviral susceptibilities. Pending the development of a suitable vaccine, antivirals will be the first line of defence in any new pandemic. Our analysis of the NAI susceptibility of 155 virus isolates from Indonesia identified eight (5%) outliers with reduced oseltamivir susceptibility with I222 mutations and a further four viruses also demonstrated loss of slow binding of oseltamivir. In comparison, even with the use of oseltamivir, only 1% of pandemic H1N1 isolates from humans have the H274Y mutation conferring oseltamivir resistance. The higher incidence of these I222 mutations that we observed is unexpected and of concern. Widespread phenotypic analysis of susceptibility of avian influenza HPAI H5N1 viruses to the NAIs needs to be carried out where the virus is endemic in poultry, to be able to respond in a timely fashion to the emergence of any strain capable of transmitting between humans.

## Materials and Methods

### Viruses

167 HPAI H5N1 avian influenza isolates from both commercial farms and the domestic sectors in SE Asia, were isolated from chickens, ducks, geese, quail and a dog. Indonesian isolates were supplied to AAHL as part of the FAO-implemented OFFLU technical project for monitoring the evolution of the HA gene. Isolates from Thailand were kindly provided by Dr Somjai Kamolsiripichaiporn, Department of Livestock Development, Bangkok, Thailand and Cambodian isolates were kindly provided by Dr Ren Theary, National Animal Health and Production Investigation Centre (NAHPIC), Department of Animal Production and Health, Phnom Penh, Cambodia. Viruses were amplified in specific pathogen free eggs under BSL3 conditions in the Diagnosis, Surveillance and Response group at CSIRO AAHL. Allantoic fluids were then gamma irradiated, prior to use in the fluorescent based NA inhibition assay.

### Chemicals and Inhibitors

Zanamivir and peramivir were kindly provided by GlaxoSmithKline (Stevenage, UK). Oseltamivir carboxylate was obtained by hydrolysis of oseltamivir phosphate (kindly provided by Dr Keith Watson Walter and Eliza Hall Institute, Australia). Dilutions of the inhibitors ranged from 0.001 nM to 10,000 nM. The fluorescent substrate 4-Methylumbelliferyl N-acetyl-α-D-neuraminic acid (MUNANA) was obtained from Sigma (Australia) or Carbosynth (Berkshire, U.K.).

### Enzyme Inhibition Assay

The MUNANA based fluorescent assay [56] was used for measuring drug inhibition. Final concentrations in the assay were 50 mM sodium acetate pH 5.5, 5 mM CaCl_2_ and 100 µM MUNANA. We used a BMG FLUOstar Optima reader with 355 nM excitation and 460 nM emission filters for all fluorescent assays. All samples were screened in duplicate against zanamivir and oseltamivir in a standard end point assay, with 30 min preincubation of inhibitor and virus followed by a 60 min reaction with MUNANA substrate. Stop solution was then added, and total fluorescent units (FU) were then measured.

Those that showed elevated IC_50_s were further evaluated in the IC_50_ kinetics assay to understand whether the elevated IC_50_s resulted from faster binding and/or faster dissociation as recently described [30], [32]. IC_50_s for end point or kinetics assays were calculated as the concentration of inhibitor resulting in a 50% reduction in FU compared to the control. The IC_50_ kinetics uses continuous real time monitoring of the enzyme reaction and two separate assays. The first assay has the standard 30 min preincubation of virus and inhibitor prior to the addition of substrate. The second assay has the simultaneous addition of virus, inhibitors and MUNANA. Fluorescence for both assays is monitored at 1 min intervals for 60 min after addition of substrate to ensure a stable signal, and IC_50_s are calculated after each 10 min interval. IC_50_s were then plotted as bar graphs for each of the 10 min time points for both assays.

### Statistical Analysis

Box and whisker plots were used to identify outliers with elevated IC_50_s [26], [27]. Means of the duplicate log_10_ IC_50_s for each sample for each drug were plotted with the box containing 50% of the samples, representing the 25% to 75% quartiles. The value between these two represents the interquartile range (IQR). Outliers were identified as mild if they were between 1.5 and 3.0 times the IQR from the 75^th^ percentile, or as extreme if they were more than 3.0 IQR from the 75^th^ percentile. The whiskers represent the 95% confidence limits.

### Sequencing

Viral RNA was extracted using the QIAamp Viral RNA Mini Kit (Qiagen, Germany). The viral NA was amplified with the SuperScript™ III One-Step RT-PCR system (Invitrogen, USA) by gene specific primers (sequences available upon request). The amplicons were gel-purified (QIAquick, Qiagen) and directly sequenced using the BigDye® Terminator (BDT) v3.1 Cycle Sequencing Kit (Applied Biosystems) according to manufacturer’s instructions. Sequencing reactions were purified with the BigDye® X-Terminator™ purification kit (Applied Biosystems) and analyzed using the Applied Biosystems 3130xl Genetic Analyzer. Full length neuraminidase gene sequences obtained by this study were submitted into GenBank with the accession numbers KC791636-KC791685, KC820950-KC820962, and KC831446-KC831550. Accession numbers for viruses named in the manuscript are listed in Table S1. Multiple sequence alignments and translation were carried out using the Bioedit program to identify potential variations associated with altered drug susceptibility [57].

## Supporting Information

Table S1 **Accession Numbers of isolates.**
(DOC)
