# Supplementary material for: I222 Neuraminidase Mutations Further Reduce Oseltamivir Susceptibility of Indonesian Clade 2.1 Highly Pathogenic Avian Influenza A(H5N1) Viruses
Source: PLoS One. 2013 Jun 11;8(6):e66105. doi: 10.1371/journal.pone.0066105 (PMC3679007; doi:10.1371/journal.pone.0066105)
Supplement: Table S1 — Accession Numbers of isolates. (DOC) [file pone.0066105.s001.doc]

**Supplementary Material**

**Supplementary Table 1 Accession Numbers of isolates**

| **Country** | **GenBank accession number** |
| --- | --- |
| **Cambodia, Clade 1.1** |  |
| A/chicken/Cambodia/CMB07.71LC3/2007 | KC820960 |
| A/duck/Cambodia/CMB07.72/2007 | KC820962 |
| A/chicken/Cambodia/CMB07.71LC4/2007 | KC820961 |
| A/chicken/Cambodia/CMB07.71LC1/2007 | KC820958 |
| A/chicken/Cambodia/CMB07.71LC2/2007 | KC820959 |
| A/duck/Cambodia/CMB06.58/2006 | KC820957 |
| A/chicken/Cambodia/CMB05.142/2005 | KC820956 |
| **Thailand, Clade 1.1** |  |
| A/chicken/Suphanburi/2509/2004 | KC820951 |
| A/chicken/Saraburi/10713/2005 | KC820952 |
| A/chicken/Pichit/606988/2006 | KC820954 |
| A/duck/Suphanburi/14376/2005 | KC820953 |
| A/chicken/Ayudhya/2057/2004 | KC820950 |
| **Indonesia, Clade 2.1** |  |
| A/chicken/Tabanan/BBVD-307/2007 | KC791657 |
| A/chicken/Bangli/BBVD-562/2007 | KC791662 |
| A/chicken/Bangli/BBVD-563/2007 | KC791663 |
| A/chicken/Pidie/BPPVRI-15/2007 | KC831496 |
| A/chicken/Tabanan/BBVD-142/2007 | KC791654 |
| A/chicken/Denpasar/BBVD-456/2007 | KC791642 |
| A/chicken/Tabanan/BBVD-107/2007 | KC791653 |
| A/chicken/Kuantan Singingi/BPPVRII-620/2007 | KC831532 |
| A/chicken/Padang Panjang/BPPVRII-272/2007 | KC831524 |
| A/chicken/Siak/BPPVRII-635/2007 | KC831534 |
| A/Muscovy duck/Magelang/BBVW-415/2007 | KC831485 |
| A/chicken/Payakumbuh/BPPVRII-307/2007 | KC831526 |
| A/chicken/West Java/Tja-31/2008 | KC831455 |
| A/chicken/West Java Tangerang/PTB6/2008 | KC831539 |
